# Protocol to obtain genetically engineered *Acetobacterium woodii* and *Eubacterium callanderi* strains

**DOI:** 10.1016/j.xpro.2025.104011

**Published:** 2025-08-04

**Authors:** Kira Sofie Baur, Barbara Rühle, Tabea Reith, Anica Krieg, Frank Robert Bengelsdorf

**Affiliations:** 1 Molecular Biology and Biotechnology of Prokaryotes, Ulm University, Albert-Einstein-Allee 11, 89069 Ulm, Germany

**Keywords:** Metabolism, Microbiology, Molecular Biology

## Abstract

We present a protocol to produce electrocompetent *Acetobacterium woodii* DSM 1030 and *Eubacterium callanderi* DSM 3468 cells. These acetogens are capable of converting CO_2_ with H_2_ and other C1 substrates into organic acids. We describe the electroporation procedure and the verification steps to prove the presence of recombinant strains. This protocol serves as a basis for the application of the genetic toolbox for these bacterial species and can be adapted to several anaerobic strains.

For complete details on the use and execution of this protocol, please refer to Leang et al.^1^

## Highlights

• Instructions to produce competent anaerobic bacteria cells
• Step-by-step protocol for genetic modification of *A. woodii* and *E. callanderi*
• Overview of common difficulties in producing recombinant strains

## Abstract (editor highlights)

Publisher’s note: Undertaking any experimental protocol requires adherence to local institutional guidelines for laboratory safety and ethics.

## Graphical abstract

**Figure:**
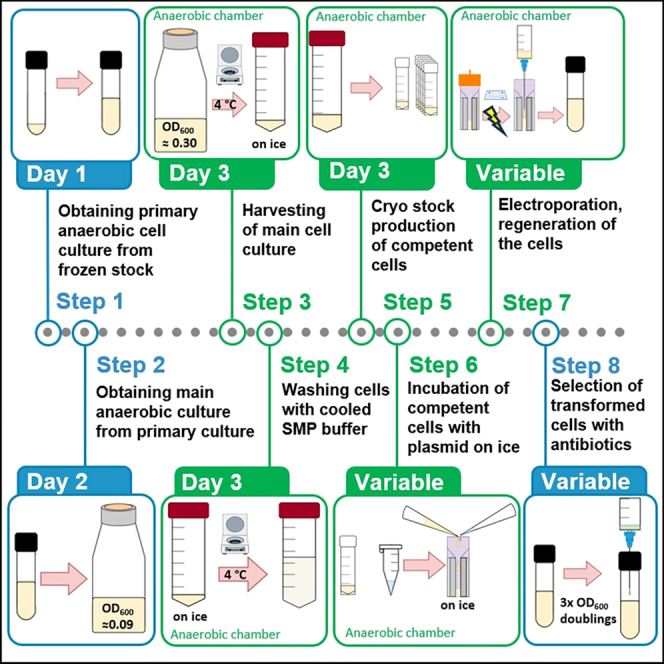

## Before you begin

The production of electrocompetent cells and subsequent transformation of *A*. *woodii* and *E*. *callanderi* cells via electroporation requires specialized equipment, including an anaerobic chamber, anaerobic serum bottles for cultivating the cells, an electroporation device, cuvettes, and a centrifuge, located within the anaerobic chamber. A suitable plasmid must also be available before cells can be transformed.

The pMTL80000 series of *Clostridium-Escherichia coli* shuttle plasmids^2^ (https://plasmidvectors.com Plasmid Vectors - Synthetic Biology Research Centre, University of Nottingham, Biodiscovery Institute, University Park, Nottingham NG7 2RD) provides several suitable options having different origins of replication (ORIs) and antibiotic resistance cassettes that are functional in acetogens.^3^ Furthermore, the *Clostridium perfringens-E. coli* shuttle vectors pJIR750 and pJIR751^4^ offer suitable features to genetically modify acetogens. Since some acetogens may harbor so far unknown antibiotic-resistance genes, the minimum inhibitory concentration has to be determined if a different antibiotic is intended to be used. The *Clostridium-E. coli* shuttle plasmids pMTL83251, pMTL83151, pMTL87151, and pJIR750 have been proven to be suitable for *A. woodii*^5^^,^^6^(this work) and pMTL83251, pMTL82251, and pJIR751 for *E. callanderi*^7^^,^^8^(this work).

This protocol uses modified DSM medium 135 (Acetobacterium medium, German Collection of Microorganisms and Cell Cultures (DSMZ), “prepare bacterial growth media” section 1a) and modified DSM 135 media (section “prepare bacterial growth media” 1b) to cultivate *A. woodii* DSM 1030 and *E. callanderi* DSM 3468. Both species were grown under strictly anaerobic conditions. Additionally, for preparing competent cells, SMP buffer (270 mM sucrose, 1 mM magnesium chloride, and 7 mM sodium phosphate) and an anti-freezing buffer (section “prepare wash and storage buffers”) are needed to store the cells. At least five days are required to carry out the full procedure described in this protocol. The time required may vary if other species are used. First, the media and buffers must be prepared and autoclaved, and the primary cell culture has to be inoculated. The next day, the main cell culture must be inoculated with additional DL-threonine (40 mM), in the anaerobic chamber, the workspace needs to be prepared. There, the main cell culture can be harvested by centrifugation, and electrocompetent cells can be obtained. The vitality of the cells needs to be checked by inoculating them in fresh media. The electroporation can be performed directly after the preparation of the electrocompetent cells. *A. woodii* and *E. callanderi* cell suspensions usually need up to two days after electroporation to double three times their optical density. Only afterwards antibiotics should be added to select for transformed cells.

### Prepare bacterial growth media

**Timing: ∼4–5 h**

1. Prepare the growth media for the bacteria.
  a. Modified DSM medium 135^5^ for the cultivation of *A. woodii.*

**Table:** Modified DSM medium 135

Reagent	Final concentration	Amount
KH_2_PO_4_	1.76 g·l^−1^	1.76 g
K_2_HPO_4_	8.44 g·l^−1^	8.44 g
NH_4_Cl	0.20 g·l^−1^	0.20 g
NaHCO_3_	10.00 g·l^−1^	10.00 g
L-cysteine-HCl	0.30 g·l^−1^	0.30 g
Na_2_S · 9 H_2_O	0.30 g·l^−1^	0.30 g
Yeast extract	3.00 g·l^−1^	3.00 g
SL-9 trace-element solution	0.20 % (v/v)	2.00 ml
Vitamin solution	0.20 % (v/v)	2.00 ml
Resazurin solution	0.10 % (v/v)	1.00 ml
Demin. water	N/A	Ad. 1.00 l
**Total**	**N/A**	**1.00 l**

Store at 4°C till color changes from yellow to pink.

    i. Add 2.00 mL trace element solution SL-9.^9^ Dissolve all components of the medium in demineralized water.
      ***Note:*** SL-9 trace element solution: Dissolve nitrilotriacetic acid, then adjust pH to 6.5 with KOH. Add minerals and adjust pH to 7.0 with KOH.
      **Table:** Trace element solution SL-9

Reagent	Final concentration	Amount
Nitrilotriacetic acid (NTA)	12.80 g·l^−1^	12.80 g
FeCl_2_ · 4 H_2_O	2.00 g·l^−1^	2.00 g
MnCl_2_ · 4 H_2_O	0.10 g·l^−1^	0.10 g
CoCl_2_ · 6 H_2_O	0.03 g·l^−1^	0.03 g
CaCl_2_ · 2 H_2_O	0.10 g·l^−1^	0.10 g
ZnCl_2_	0.10 g·l^−1^	0.10 g
CuCl_2_	0.02 g·l^−1^	0.02 g
H_3_BO_3_	0.01 g·l^−1^	0.01 g
Na_2_MoO_4_ · 2 H_2_O	0.03 g·l^−1^	0.03 g
NiCl_2_ · 6 H_2_O	0.10 g·l^−1^	0.10 g
NaCl	1.00 g·l^−1^	1.00 g
Na_2_SeO_3_ · 5 H_2_O	0.03 g·l^−1^	0.03 g
Na_2_WO_4_ · 2 H_2_O	0.04 g·l^−1^	0.04 g
Demin. water	N/A	Ad. 1.00 l
**Total**	**N/A**	**1.00 l**

Store at −20°C up to five years.
    ii. Add 2.00 mL vitamin solution.^5^

**Table:** Vitamin solution

Reagent	Final concentration	Amount
Biotin	0.025 g·l^−1^	0.025 g
Folic acid	0.025 g·l^−1^	0.025 g
Pyridoxine-HCl	0.050 g·l^−1^	0.050 g
Thiamine-HCl · 2 H_2_O	0.050 g·l^−1^	0.050 g
Riboflavin	0.050 g·l^−1^	0.050 g
Nicotinic acid	0.050 g·l^−1^	0.050 g
D-Ca-pantothenate	0.050 g·l^−1^	0.050 g
Cyanocobalamine	0.025 g·l^−1^	0.025 g
α-Aminobenzoic acid	0.050 g·l^−1^	0.050 g
Lipoic acid	0.025 g·l^−1^	0.025 g
Demin. water	N/A	Ad. 1.00 l
**Total**	**N/A**	**1.00 l**

Store at −20°C up to five years.
    iii. Add 1.00 mL resazurin solution (1.00 g·L^−1^).
    iv. Dissolve all components of the medium in demineralized water.
  b. Modified DSM medium 135^7^ for cultivating *E. callanderi*:

**Table:** Modified DSM medium 135

Reagent	Final concentration	Amount
KH_2_PO_4_	1.76 g·l^−1^	1.76 g
K_2_HPO_4_	8.44 g·l^−1^	8.44 g
NH_4_Cl	1.00 g·l^−1^	1.00 g
NaHCO_3_	6.00 g·l^−1^	6.00 g
L-cysteine-HCl	0.44 g·l^−1^	0.44 g
NaCl	3.00 g·l^−1^	3.00 g
Yeast extract	2.00 g·l^−1^	2.00 g
SL-9 trace-element solution	0.10 % (v/v)	1.00 ml
Selenite-tungstate solution	0.10 % (v/v)	1.00 ml
Vitamin solution	0.10 % (v/v)	1.00 ml
Resazurin solution	0.10 % (v/v)	1.00 ml
Demin. water	N/A	Ad. 1.00 l
**Total**	**N/A**	**1.00 l**

Store at 4°C till color changes from yellow to pink.

    i. Add 1.00 mL trace element solution SL-9^9^ and 1.00 ml selenite-tungstate solution.^9^

**Table:** Selenite-tungstate solution

Reagent	Final concentration	Amount
NaOH	0.40 g·l^−1^	0.40 g
Na_2_SeO_3_ · 5 H_2_O	6.00 mg·l^−1^	6.00 mg
Na_2_WO_4_ · 2 H_2_O	8.00 mg·l^−1^	8.00 mg
Demin. water	N/A	Ad. 1.00 l
**Total**	**N/A**	**1.00 l**

Store at −20°C up to five years.
    ii. Last add 1.00 ml vitamin solution DSM 141.

**Table:** Vitamin solution DSM 141

Reagent	Final concentration	Amount
Biotin	2.00 mg·l^−1^	2.00 mg
Folic acid	2.00 mg·l^−1^	2.00 mg
Pyridoxine-HCl	10.00 mg·l^−1^	10.00 mg
Thiamine-HCl · 2 H_2_O	5.00 mg·l^−1^	5.00 mg
Riboflavin	5.00 mg·l^−1^	5.00 mg
Nicotinic acid	5.00 mg·l^−1^	5.00 mg
D-Ca-pantothenate	5.00 mg·l^−1^	5.00 mg
Cyanocobalamine	0.10 mg·l^−1^	0.10 mg
α-aminobenzoic acid	5.00 mg·l^−1^	5.00 mg
Lipoic acid	5.00 mg·l^−1^	5.00 mg·l^−1^
Demin. water	N/A	Ad. 1.00 l
**Total**	**N/A**	**1.00 l**

Store at −20°C up to five years.
    iii. Add 1.00 ml of resazurin solution (1.00 g·l^−1^).
    iv. Dissolve all components of the medium in demineralized water.
2. Fill the media in hungates (5 ml per hungate) or bottles (50 mL per bottle). The cap of the hungate or bottle contains two parts, a rubber stopper and an autoclavable screw cap. First, place the rubber stopper in the opening and then screw the cap on it.
3. Use a vacuum pump to generate underpressure in the vessels and refill with an 80% N_2_ and 20% CO_2_ gas atmosphere, repeat this seven times. Then, puncture the rubber stopper with a cannula to release overpressure.

***Note:*** Alternatively, cook the media for 20 min. Afterward, cool it on ice, while flushing it with N_2_. If the medium is cooled down, it can be aliquoted into hungates or bottles in the anaerobic chamber.

4. Autoclave the media for 15 min at 121°C and 1.2 bar.

***Note:*** Check first if your bottle can withstand this pressure.

5. Add sterile, anaerobic MgSO_4_ and fructose solution using syringes to reach a final concentration of 1.5 mM MgSO_4_ and 45 mM fructose.
6. Store the media, fructose solution, and MgSO_4_ solution at room temperature.

***Note:*** If you store them at 4°C, incubate them at room temperature or the desired cultivation temperature before using them.

7. Aliquot the trace element solution SL-9,^9^ vitamin solution,^10^ selenite-tungstate solution,^9^ and vitamin solution DSM 141 in 50 mL aliquots and freeze them at −20°C.

### Prepare wash and storage buffers

**Timing: ∼2 h**

8. Prepare SMP buffer.
  a. SMP buffer contains 92.42 g·l^−1^sucrose, 0.204 g·l^−1^ MgCl_2_·6H_2_O and 0.840 g·l^−1^ NaH_2_PO_4_.
  b. Adjust the pH to a value of 6.
  c. Cook the buffer for 20 min.
  d. Afterwards, cool it on ice, while flushing the buffer with N_2_.
  e. Finally, refill the buffer with anaerobic water to the desired volume in an anaerobic chamber.
9. Prepare an anti-freezing buffer by mixing SMP buffer with 20% anaerobic, sterile dimethyl sulfoxide under anaerobic conditions.
10. Store SMP- and anti-freezing buffer at 4°C.

### Workspace preparation

**Timing: ∼30 min**

**Figure 1:**
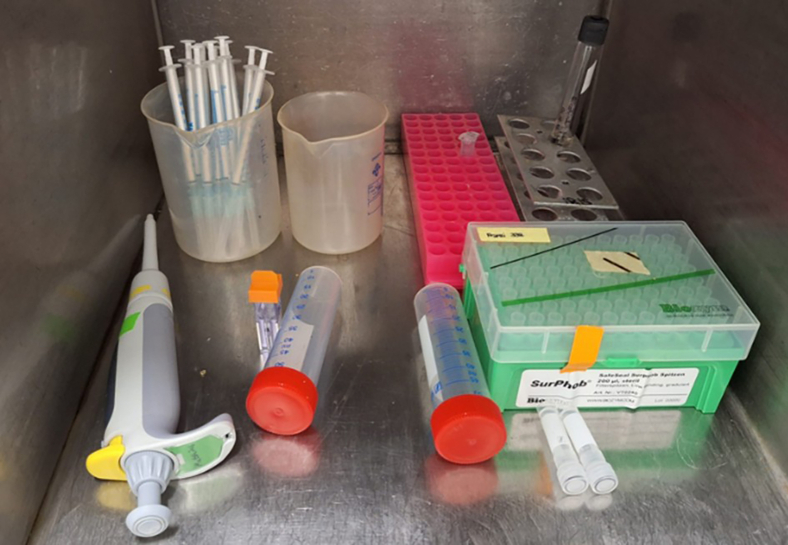
Prepared materials in the transfer gate of the anaerobic chamber for the production of competent cells and their transformation

11. The day before preparing competent cells and the electroporation procedure:

Transfer all needed materials and devices into the anaerobic chamber to degas the remaining oxygen in the devices (Figure 1).

**CRITICAL:** Adherent oxygen in plastic devices may promote lethal effects on anaerobic bacteria.

### Isolation of the plasmid from *E*. *coli*

**Timing: ∼30 min**

12. Inoculate *E. coli* strains that harbor the plasmid of interest with respective antibiotics in lysogeny broth media.^11^
13. Incubate while shaking at 37°C and 130 rpm overnight.
14. Harvest the cells.
15. Isolate the plasmid with the Zyppy Plasmid Miniprep Kit (Zymo Research) according to the manufacturer’s instructions.

***Alternatives:*** Alternatively, the plasmid can be isolated with the Monarch Plasmid Miniprep Kit.

16. Determine plasmid DNA concentration.

***Note:*** Choose an *E. coli* strain carrying no DNA methyltransferases, such as *E. coli* DH5a or *E. coli* SCS110. Bacteria tend to methylate their own DNA in a specific way and harbor respective restriction-modification systems (RM)^1^^,^^12^ as a defense mechanism, which protects the organisms from foreign DNA. There is not much known about restriction modification systems in *A. woodii* and *E. limosum*.^13^ However, unmethylated DNA from *E. coli* DH5α or *E. coli* SCS110 is accepted by both organisms.

## Key resources table

**Table:** 

REAGENT or RESOURCE	SOURCE	IDENTIFIER
**Bacterial and virus strains**		

*A. woodii ΔpyrE ΔlctBCD* *A. woodii* DSM1030	Mook et al.^6^ German Collection of Microorganisms and Cell Cultures (DSMZ)	Awo_c00860, Locus Tag 16S rDNA
*E. callanderi* DSM 3468	German Collection of Microorganisms and Cell Cultures (DSMZ)	EUCMar_02330 Locus Tag 16S rDNA

**Chemicals, peptides, and recombinant proteins**		

α-Aminobenzoic acid	Fluka	150-13-0
Ammonium chloride	Carl Roth	12125-02-9
Biotin	Sigma	58-85-5
Boric acid	Merck	10043-35-3
Calcium chloride dihydrate	Merck	10035-04-8
Cobalt chloride hexahydrate	Merck	7791-13-1
Copper (II) chloride hexahydrate	Merck	10125-13-0
Cyanocobalamin	Fluka	68-19-9
D-calcium pantothenate	Merck	137-08-6
Dimethyl sulfoxide	Carl Roth	67-68-5
Dipotassium hydrogen carbonate	Carl Roth	298-14-6
Folic acid	Sigma	59-30-3
Fructose	Carl Roth	57-48-7
Iron (II) chloride tetrahydrate	Merck	13478-10-9
L-cysteine hydrochloric acid monohydrate	Merck	7048-04-6
Lipoic acid	Fluka	1077-28-7
Magnesium chloride hexahydrate	Sigma	7791-18-6
Magnesium sulfate heptahydrate	Sigma	10034-99-8
Manganese (II) chloride dihydrate	Merck	20603-88-7
Nickel chloride hexahydrate	Sigma	7718-54-9
Nicotic acid	AppliChem	59-67-6
Nitrilotriacetic acid	Fluka	139-13-9
Potassium bicarbonate	AppliChem	298-14-6
Potassium phosphate dibasic	Sigma	7758-11-4
Potassium phosphate monobasic	Sigma	7778-77-0
Pyridoxine hydrochloric acid	Fluka	58-56-0
Riboflavin	Supelco	83-88-5
Saccharose	Carl Roth	57-50-1
Sodium bicarbonate	Carl Roth	144-55-8
Sodium chloride	Carl Roth	7647-14-5
Sodium dihydrogen phosphate	Sigma	13472-35-0
Sodium dioxido(dioxo)molybdenum dihydrate	Merck	10102-40-6
Sodium hydroxide	Carl Roth	1310-73-2
Sodium selenite pentahydrate	Merck	26970-82-1
Sodium sulfide nonahydrate	Merck	1313-84-4
Sodium tungstate dihydrate	Merck	10213-10-2
Thiamine hydrochloric acid dihydrate	Merck	67-03-8
Yeast extract	Batco	REF: 212750
Zinc chloride	Fluka	7646-85-7

**Recombinant DNA**		

pMTL87151	Plasmid Vectors - Synthetic Biology Research Centre University of Nottingham Biodiscovery Institute University Park Nottingham NG7 2RD	N/A
pMTL83251	Plasmid Vectors - Synthetic Biology Research Centre University of Nottingham Biodiscovery Institute University Park Nottingham NG7 2RD	N/A
pJIR751	Bannam and Rood^4^	N/A

**Software and algorithms**		

Draw.io()	Graphic created using Draw.io 2005-2023 JGraph Ltd., Artisans' House, 7 Queensbridge, NN4 7BF, Northampton, England. Company #04051179.	N/A

**Other**		

Anaerobic chamber	custom-made	
N_2_ + H_2_ (95% nitrogen with 5% hydrogen)	MTI Industriegase AG (Neu-Ulm, Germany)	7727-37-9 (CAS N2) + 1333-74-0 (CAS H2)
N_2_ + CO_2_ (80% nitrogen with 20% carbon dioxide)	MTI Industriegase AG (Neu-Ulm, Germany)	7727-37-9 (CAS N2) + 124-38-9 (CAS CO2)
N_2_ (100% nitrogen)	MTI Industriegase AG (Neu-Ulm, Germany)	7727-37-9
Threaded bottle DURAN pressure+ protect+, 500 ml	DWK Life Sciences	500 mL
Rubber stoppers	DWK Life Sciences	GL 45
Screw caps with bore	DWK Life Sciences	GL 45
Cooling centrifuge	Hettich	Universal 320R
Falcon tubes	Sarstedt AG	62.547.205
Gene Pulser Xcell Electroporation Systems	Bio-Rad	#1652660
Electroporation cuvettes 1 mm	Biozym Scientific GmbH	748010
Cryo-tubes	Sarstedt AG	72.694.600
Syringes	Ersta	1 mL, 2 mL, 5 mL
Cannulas	BD Microlance	0.8 × 40 mm
Hungates	Bellco	2047–00125
Hungate photometer	Thermo Scientific	Genesys30
Zyppy Plasmid Miniprep Kit	The Epigenetics Company	ZRC178558

## Step-by-step method details

### Preparation of electrocompetent cells using the example of *A. woodii*

**Timing: ∼18 h (split across multiple days)**

***Note:*** The same protocol can be used for *E. callanderi*.

Day 1 ∼5 pm:

1. Inoculate the *A. woodii* main cell culture in 100 mL DSM 135 mod. media^5^ with 40 mM DL-threonine and 45 mM fructose to an OD_600_ of 0.09 ± 0.05.
2. Incubate overnight at 30°C.

**Figure 2:**
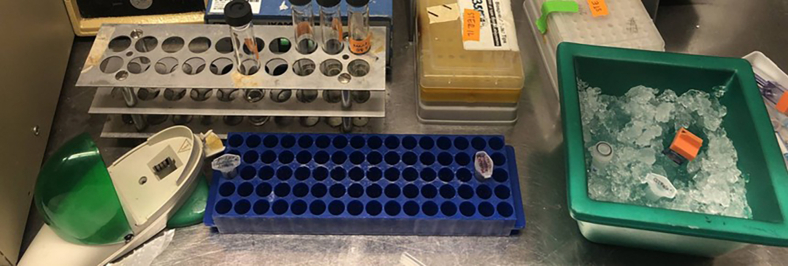
Prepared workspace in the anaerobic chamber with competent cell suspension in a cryotube, an electroporation cuvette, and plasmid DNA on ice

**Figure 3:**
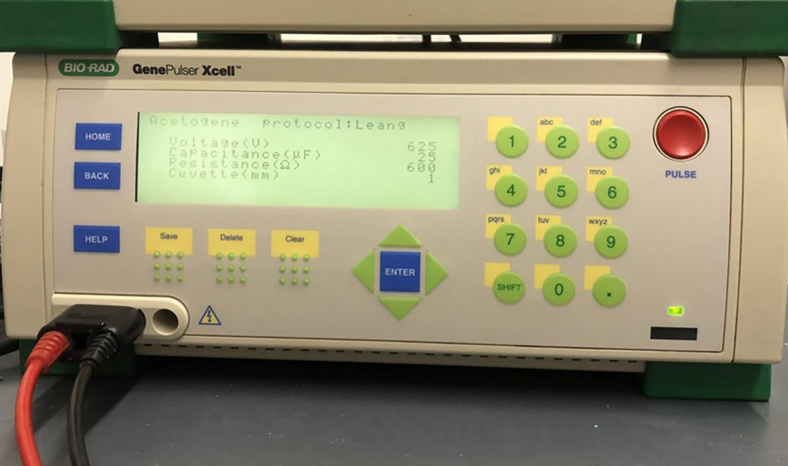
Bio-Rad GenePulser Xcell with set parameters for the electroporation

**Figure 4:**
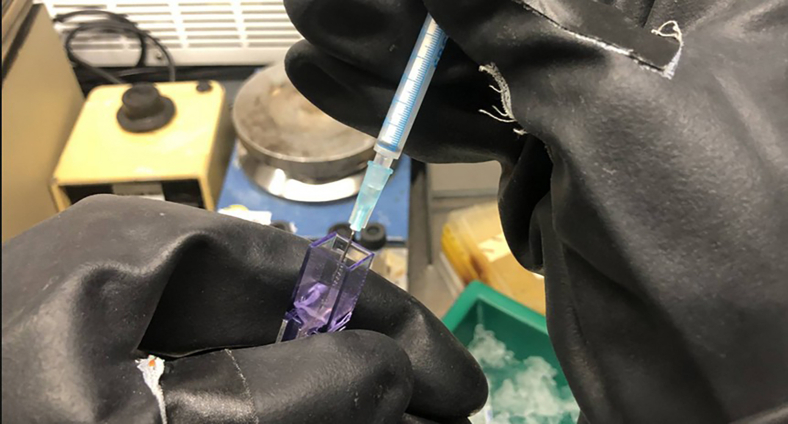
Transfer of electroporated cells from electroporation cuvette into fresh media with a syringe

Day 2 ∼9 am.

3. *A. woodii* main cell culture should have reached an OD_600_ of around 0.3.
4. Put the bottle of this cell suspension on ice.
5. Transfer the cooled *A. woodii* cell suspension, the pre-cooled SMP, and anti-freezing buffer into the anaerobic chamber.
6. Store the buffers and the cell suspension on ice.
7. Divide the *A. woodii* cell suspension into 50 mL falcon tubes and harvest the cells by centrifugation at 4°C, 7,690 × *g* for 10 min.
8. Discard supernatant.
9. Wash the cells twice with 20 mL cooled SMP buffer and centrifuge at 4°C, 7,690 × *g* for 10 min respectively.
10. Discard supernatant.
11. Suspend the cell pellet in each falcon in 720 μL cooled anti-freezing buffer.
12. Transfer the cell suspension in cryo-tubes and store at −80°C.

### Transformation of electrocompetent cells using the example of *A. woodii*

**Timing: ∼2 h**

***Note:*** The same protocol can be used for *E. callanderi*.

***Note:*** The following steps need to be performed in an anaerobic chamber.

13. Mix 3–5 μg plasmid DNA with 25 μL of electrocompetent cells in a pre-cooled 1 mm electroporation cuvette (Figure 2).
14. Incubate the cell plasmid mixture on ice for 5 min.
15. Put the electroporation cuvette in a Shockpod cuvette chamber and apply current with the conditions: 625 V, 25 μF, 600 Ω (Figure 3).
16. Transfer the cells immediately into 5 mL fresh medium (Figure 4) without antibiotics.
17. Recover the *A. woodii* cells at 30 °C in 5 mL modified DSM 135 medium.
18. Monitor the OD_600_ of the electroporated cells.
19. Induce the cells after two doublings by adding sufficient amounts of the appropriate antibiotics with a syringe to select the recombinant strain.

***Note:*** Recommended antibiotics for working with *A. woodii* are clarithromycin (5 μg mL^−1^) and thiamphenicol (15 μg mL^−1^). The antibiotic stocks do not necessarily have to be anaerobic.

***Note:*** Bring a waste vessel into the anaerobic chamber. Store an adhesive tape in the anaerobic chamber to fix potential holes in the gloves. If you puncture yourself with a syringe through the glove, follow the first aid instructions of your lab.

## Expected outcomes

This protocol describes a validated method based on Leang et al., 2013^1^ to produce electrocompetent cells of different anaerobic species. The final results of this protocol are recombinant strains of anaerobic bacteria such as *A. woodii* or *E. callanderi* (Table 1), which need to be verified by plasmid isolation and restriction digest or PCR amplification (Figures S1–S7). Since plasmids carry an antibiotic resistance gene, the recombinant strain must grow in the presence of the specific antibiotic and show at least three doublings. The described protocol was successfully used to obtain the recombinant strains listed in Table 2.

**Table 1:** Selection of performed transformations with variations of the parameters plasmid DNA, plasmid sizes, ORI, and used plasmid DNA masses

Plasmid	ORI	Plasmid size (bp)	Used plasmid mass (ng)	Antibiotic resistance genes	Organism	Number of hungate tubes inoculated with cell suspension	Number of cell suspensions growing in the presence of antibiotics that have been positively validated by proving presence of plasmid DNA
pMTL87151 (Figure S5)	pUB110	4,589	3,000; 4,000; 5,000	*catP*	*A. woodii ΔpyrE ΔlctBCD*^6^	3; 2; 2	3; 2; 2
pMTL87151 (Figure S4)	pUB110	4,589	3,000	*catP*	*A. woodii ΔpyrE ΔlctBCD* [pMTL83251]	2	2
∗pMTL83251^2^ (Figure S6)	pCB102	5,493	3,000	*ermB*	*A. woodii ΔpyrE ΔlctBCD*^6^	2	2
pMTL83251^2^	pCB102	5,493	4,000	*ermB*	*A. woodii ΔpyrE ΔlctBCD* [pMTL87151] (Baur, unpublished)	2	2
pMTL871ksb_Ppta-ack_act_PackA-theo_pfl (this study) (Figure S7)	pUB110	7,954	3,000	*catP*	*A. woodii ΔpyrE ΔlctBCD*^6^	2	2
pMTL871ksb_Ppta-ack_act_PackA-theo_pfl (this study) (Figure S7)	pUB110	7,954	3,000	*catP*	*A. woodii ΔpyrE ΔlctBCD* [pMTL83251_PlctA_ldhD-NFP]	2	2
pMTL871ksb_Ppta-ack_act_PackA-theo_pfl (this study) (Figure S7)	pUB110	7,954	3,000	*catP*	*A. woodii ΔpyrE ΔlctBCD* [pMTL83251_PbgaL_ldhD-NFP]^6^	2	2
MPD21^14^ (Figure S8)	pCD6	9,315	3,000; 4,000	*catP*	*A. woodii ΔpyrE ΔlctBCD*^6^	2; 2	2; 2
MPD23_PbgaL_ldhD-LM (this study) (Figure S3)	pCD6	11,696	3,000; 4,000; 5,000	*catP*	*A. woodii ΔpyrE ΔlctBCD ΔpheA* (Baur, unpublished)	4; 4; 2	0; 4; 0
pJIR751^4^ (Figure S2)	pIP404	5,954	3,500	*ermB*	*E. callanderi* DSM 3468^8^	3	3
pJIR751_PbgaL_FAST (Flaiz, unpublished) (Figure S1)	pIP404	8,061	3,500	*ermB*	*E. callanderi* DSM 3468^8^	2	2

The ∗-marked plasmid pMTL83251 is a suitable control to check electrocompetent *A. woodii* or *E. callanderi* cells, and proper implementation of the transformation via electroporation.

**Table 2:** Successfully performed transformations following this protocol with different species

Organism	Existing genetic modifications
*A. woodii* DSM 1030^T^	• plasmid-based expression of the acetone production operon from *Clostridium acetobutylicum* ATCC 824 including the thiolase A (ThlA), the acetoacetyl-CoA:acetate/butyrate CoA transferase (CtfA/CtfB), and the acetoacetate decarboxylase (Adc)^5^ • plasmid-based expression of the arginine deiminase pathway genes from *Clostridium autoethanogenum* DSM 10061^15^ • plasmid-based expression of an isobutanol synthesis pathway consisting of the ketoisovalerate ferredoxin oxidoreductase (Kor) from *Clostridium thermocellum* DSM 1313 and the bifunctional aldehyde/alcohol dehydrogenase (AdhE2) from *C. acetobutylicum* ATCC 824^16^ • plasmid-based expression of an isobutanol synthesis pathway consisting of ketoisovalerate decarboxylase from *Lactococcus lactis* and alcohol dehydrogenase from *Corynebacterium glutamicum* DSM 20300^16^ • plasmid-based expression of hcs cluster from *C. carboxidivorans* P7 DSM15243 for conversion of acetyl-CoA to butyryl-CoA by reverse β-oxidation^17^ • improvement of the acetone production in A. woodii via the plasmid-based expression of the thiolase A (ThlA) from *Clostridium kluyveri* DSM 555, and the acetoacetyl-CoA:acetate/butyrate CoA transferase (CtfA/CtfB), and the acetoacetate decarboxylase (Adc) from *C. acetobutylicum* ATCC 824^18^ • improvement of the acetone production in *A. woodii* via the plasmid-based expression of the acetoacetyl-CoA:acetate/butyrate CoA transferase (CtfA/CtfB) from *C. aceticum* DSM 1496, and the thiolase A (ThlA) and the acetoacetate decarboxylase (Adc) from *C. acetobutylicum* ATCC 824^18^ • improvement of the acetone production in *A. woodii* via the plasmid-based expression of the thiolase A (ThlA) from *Clostridium kluyveri* DSM 555, the acetoacetyl-CoA:acetate/butyrate CoA transferase (CtfA/CtfB) from *C. aceticum* DSM 1496, and the acetoacetate decarboxylase (Adc) from *C. acetobutylicum* ATCC 824^19^ • improvement of the acetone production in *A. woodii* via the plasmid-based expression of the thiolase A (ThlA) and the acetoacetyl-CoA:acetate/butyrate CoA transferase (CtfA/CtfB) from *C. scatologenes* DSM 757, and the acetoacetate decarboxylase (Adc) from *C. acetobutylicum* ATCC 824^19^ • improvement of the acetone production in *A. woodii* via the plasmid-based expression of the thiolase A (ThlA) from *C. scatologenes* DSM 757 and the acetoacetyl-CoA:acetate/butyrate CoA transferase (CtfA/CtfB), and the acetoacetate decarboxylase (Adc) from *C. acetobutylicum* ATCC 824^19^ • improvement of the isopropanol production in A. woodii via the plasmid-based expression of the acetone production operon from *Clostridium acetobutylicum* ATCC 824 including the thiolase A (ThlA), the acetoacetyl-CoA:acetate/butyrate CoA transferase (CtfA/CtfB), and the acetoacetate decarboxylase (Adc) and the secondary alcohol dehydrogenase (SadH) of *Clostridium beijerinckii* DSM 6423^10^ • improvement of the isopropanol production in *A. woodii* via the plasmid-based expression of the acetone production operon from Clostridium acetobutylicum ATCC 824 including the thiolase A (ThlA), the acetoacetyl-CoA:acetate/butyrate CoA transferase (CtfA/CtfB), and the acetoacetate decarboxylase (Adc) and the secondary alcohol dehydrogenase (SadH) of *Clostridium ljungdahlii* DSM 13528^10^ • plasmid-based expression of a synthetic 3-hydroxybutyrate pathway including the thiolase A (ThlA), the 3-hydroxy butyryl-CoA dehydrogenase gene (Hbd), and the crotonase (Crt) from *C. scatologenes* ATCC 25775 as well as the (R)-enoyl-CoA hydratase gene (PhaJ) and polyhydroxyalkanoate (PHA) synthase (PhaC/ PhaE) from *C. acetireducens* DSM 10703^10^
*Clostridium coskatii* ATCC PTA-10522	• plasmid-based expression of the genes expressing gene products for polyhydroxy butyrate (PHB) synthesis originating from *Cupriavidus necator* DSM 428^20^ • plasmid-based expression of the genes required for PHB synthesis originating from *Burkholderia thailandensis* DSM 13276^20^ • plasmid-based expression of a synthetic 3-hydroxybutyrate pathway including the thiolase A (ThlA) and the acetoacetyl-CoA:acetate/butyrate CoA transferase (CtfA/CtfB) originating from *C. acetobutylicum* ATCC 824 and the 3-hydroxybutyrate dehydrogenase (BdhA) from Clostrioides difficile DSM 27543^20^ • plasmid-based expression of a synthetic 3-hydroxybutyrate pathway including the thiolase A (ThlA), the 3-hydroxy butyryl-CoA dehydrogenase gene (Hbd), and the crotonase (Crt) from *C. scatologenes* ATCC 25775 as well as the (R)-enoyl-CoA hydratase gene (PhaJ) and polyhydroxyalkanoate (PHA) synthase (PhaC/ PhaE) from *C. acetireducens* DSM 10703^20^
*Clostridium ljungdahlii* DSM 13583^T^	• plasmid-based expression of the genes needed for PHB synthesis originating from *Cupriavidus necator* DSM 428^20^ • plasmid-based expression of the genes required for PHB synthesis originating from *Burkholderia thailandensis* DSM 13276^20^ • plasmid-based expression of a synthetic 3-hydroxybutyrate pathway including the thiolase A (ThlA) and the acetoacetyl-CoA:acetate/butyrate CoA transferase (CtfA/CtfB) originating from *C. acetobutylicum* ATCC 824 and a gene encoding the hydroxybutyrate dehydrogenase (BdhA) from Clostrioides difficile DSM 27543^20^ • plasmid-based expression of a synthetic 3-hydroxybutyrate pathway including the thiolase A (ThlA), the 3-hydroxy butyryl-CoA dehydrogenase (Hbd), and the crotonase from *C. scatologenes* ATCC 25775 as well as the genes encoding the (R)-enoyl-CoA hydratase (PhaJ) and PHA synthase (PhaC/ PhaE) from *C. acetireducens* DSM 10703^20^ • plasmid-based expression of an isobutanol synthesis pathway consisting of ketoisovalerate ferredoxin oxidoreductase (Kor) from *Clostridium thermocellum* and bifunctional aldehyde/alcohol dehydrogenase (AdhE2) from *C. acetobutylicum*^16^ • plasmid-based expression of an isobutanol synthesis pathway consisting of ketoisovalerate decarboxylase from *Lactococcus lactis* and alcohol dehydrogenase from *Corynebacterium glutamicum*^16^
*A. woodii ΔpyrE ΔlctBCD*	• plasmid-based expression of the D-lactate dehydrogenase originating from *Leuconostoc mesenteroides*^6^ • plasmid-based expression of a fluorescence-activating and absorption-shifting tag (FAST)^6^ • plasmid-based expression of a tandem fluorescence-activating and absorption-shifting tag (FAST2)-tagged D-lactate dehydrogenase originating from *L. mesenteroides*^6^
*Eubacterium limosum* B2 (not publicly available)	• plasmid-based expression of FAST^7^^,^^8^ • plasmid-based expression of FAST2^7^ • plasmid-based expression of the bifunctional aldehyde/alcohol dehydrogenase (AdhE2) FAST-tagged and non-FAST-tagged from *C. acetobutylicum*^7^ • plasmid-based expression of the acetone production operon including thiolase A (ThlA), the acetoacetyl-CoA:acetate/butyrate CoA transferase (CtfA/CtfB), and the acetoacetate decarboxylase (Adc) originating from *C. acetobutylicum* ATCC 824^7^ • plasmid-based expression of the FAST-tagged acetoacetate decarboxylase (Adc) originating from *C. acetobutylicum* ATCC 824^7^
*E. limosum* DSM 20543^T^	• plasmid-based expression of FAST^8^
*Eubacterium callanderi* DSM 3662^T^	• plasmid-based expression of FAST^8^
*Eubacterium callanderi* KIST612 (not publicly available)	• plasmid-based expression of FAST^8^
*Eubacterium callanderi* DSM 2593	• plasmid-based expression of FAST^8^
*Eubacterium callanderi* DSM 2594	• plasmid-based expression of FAST^8^
*Eubacterium maltosivorans* DSM 20517	• plasmid-based expression of FAST^8^

## Limitations

This protocol was successfully used to perform electroporations of cells with plasmids, ranging from 4,000 to 12,000 bp (Tables 1 and 2). DNA masses of plasmids between 3.0 and 5.0 μg DNA usually result in a successful transformation. The recovery time of the cells after transformation depends on the species, the quality of the produced competent cells, and the size of the plasmid DNA in base pairs. Slow-growing species and transformations involving large plasmids may require extended regeneration time.

Another limitation is the cultivation of *A. woodii* and *E. callanderi* on a plate. These organisms do not grow on plates in the gas atmosphere of our anaerobic chamber (95% N_2_ + 5% H_2_). An option is to cultivate them on slant-poured agar, in hungates or anaerobic bottles to change the gas atmosphere around the poured agar. The colonies only grow on the thin edge areas of the agar, which is why single colonies are very rare and a selection from solid medium is difficult and laborious. In this protocol, *A. woodii* and *E. callanderi* cells are grown in closed hungate tubes after electro-pulsing to avoid contamination.

Humphreys et al.^21^ reached promising transformation efficiencies in their study with *E. callanderi*, formerly known as *Butyribacterium methylotrophicum*, using a similar protocol. They used 1.0 μg plasmid DNA of pMTL83151, pMTL84151, and pJRHN1. With pMTL83151 they reached a transformation efficiency of 18.0 ± 2.5 cell forming units (CFU)/μg DNA, followed by pMTL84151 at 305.0 ± 38.0 CFU/μg DNA and pJRHN1 with by far the highest transformation efficiency of 6.0 · 10^5^ ± 3.0 · 10^4^ CFU/ μg DNA.^21^ Leang and colleagues optimized their protocol for the transformation of *C. ljungdahlii* DSM 13528 reaching transformation efficiencies of 1.1 ± 0.1 transformants/μg DNA with the vector pCL1, 14.9 ± 4.9 transformants/μg DNA with pQexp, 1.7 · 10^4^ ± 0.6 · 10^4^ transformants/μg DNA with pCL2, 3.8 · 10^3^ ± 0.2 · 10^3^ transformants/μg DNA with the vector pMTL82151 and 3.1 · 10^3^ ± 1.8 · 10^3^ transformants/μg DNA with the vector pMTL83151.^1^ The optimizations to increase the transformation efficiency comprise lowering the pH of the SMP buffer from 7.4 to 6, harvesting the cell to prepare competent cells at an OD_600_ value of 0.2–0.3 instead of 0.3–0.7, and increasing the cell density in the final electrocompetent cell suspension.^1^ The work of Humphreys and colleagues and Leang and colleagues shows that choosing a vector with a suitable ORI positively affects transformation efficiency.

## Troubleshooting

### Problem 1

Arcing with a cracking noise in the cuvette chamber during the setting of the electric pulse while transforming the electrocompetent cells (“transformation of electrocompetent cells using the example of A. woodii,” step 15).

### Potential solution

The DNA was diluted in a buffer with too high salt concentration. A high salt concentration can lead to a short circuit during electroporation. A solution is to reduce the salts in the buffer or to replace the buffer with water before it is added to the cells.

### Problem 2

Low transformation yield (“transformation of electrocompetent cells using the example of A. woodii,” steps 13–19).

### Potential solution

• The transformation efficiency of the electrocompetent cells can be diminished if the cells and the buffers are not cooled during the production of the competent cells and the transformations. Ensure that the cells are always cooled during the process!
• It is important to check the restriction-modification system (RM system) of other species that should be transformed, as well as the *E. coli* strain in which the plasmid was constructed, to carry out a successful transformation. Both the RM systems of the organism to be transformed and the *E. coli* strain should match.
• Adapting the electroporation parameters (e.g. voltage) can be a solution.
• It is possible to freeze and thaw the competent cells multiple times, but the transformation efficiency decreases with the number of thaw cycles. Even longer storage times can reduce the transformation efficiency.
• Too little DNA mass has been used.
• Avoid using low concentrations of plasmid DNA that would result in a large volume of plasmid DNA solution added. Too high dilution of the cell suspension can cause a lower transformation efficiency. Avoid DNA solution volumes of more than 10 μL.

## Resource availability

### Lead contact

Further information should be directed to and will be fulfilled by the lead contact, Kira Sofie Baur (kira.baur@uni-ulm.de).

### Technical contact

Questions about the technical specifics of performing the protocol should be directed to and will be answered by the technical contact, Kira Sofie Baur (kira.baur@uni-ulm.de).

### Materials availability

This protocol generated a collection of recombinant *A. woodii* strains with resistances against clarithromycin and thiamphenicol.

### Data and code availability

This study did not generate datasets or codes.

## Acknowledgments

The authors acknowledge Franzsiska Höfele and Felix M. Wagenblast for proofreading.

This study was funded by the 10.13039/501100001659German Research Foundation DFG (priority program InterZell [SPP2170], project CaproSyn, project no. 427864786).

## Author contributions

K.S.B. drafted the text for this manuscript, illustrated the graphical abstract using draw.io, and performed the *A. woodii* transformations shown. B.R. performed the *E. callanderi* transformations shown. A.K. and T.R. performed a transformation of *E. callanderi* cells to illustrate the text with photos and looked up the identifier of the used materials. F.R.B. supervised the study.

## Declaration of interests

The authors declare no competing interests.
