# Supplementary material for: Protocol to obtain genetically engineered Acetobacterium woodii and Eubacterium callanderi strains
Source: STAR Protoc. 2025 Aug 4;6(3):104011. doi: 10.1016/j.xpro.2025.104011 (PMC12345282; doi:10.1016/j.xpro.2025.104011)
Supplement: Document S1. Figures S1–S8 [file mmc1.pdf]

Supplementary

Exemplary verification of the transformed *A. woodii* and *E. callanderi* strains:

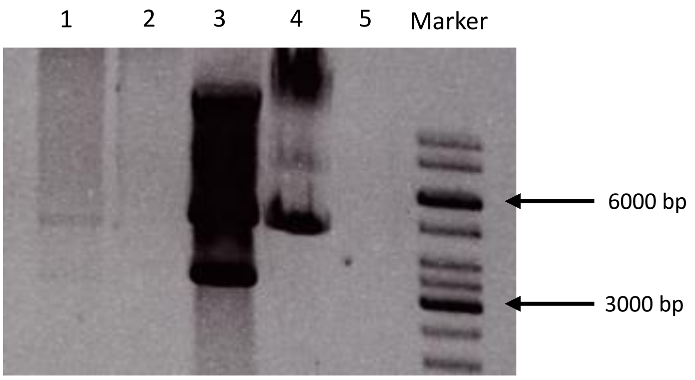

Figure S1: The Agarose gel shows DNA fragments obtained after restriction digestion of the plasmid pJIR751_P_{bgaL}_FAST to verify the presence of pJIR751_P_{bgaL}_FAST, related to "Expected Outcomes", Table 2. Lane 1: pJIR751_P_{bgaL}_FAST isolated from *E. callanderi* digested with BamHI and ScaI. Lane 2: Empty. Lane 3: positive control, pJIR751_P_{bgaL}_FAST isolated from *E. coli* DH5α, digested with BamHI and ScaI. Fragments: 5039 bp and 3022 bp (expected fragments: 5039 bp and 3022 bp). Marker: GeneRuler 1 kb Thermo Fisher.

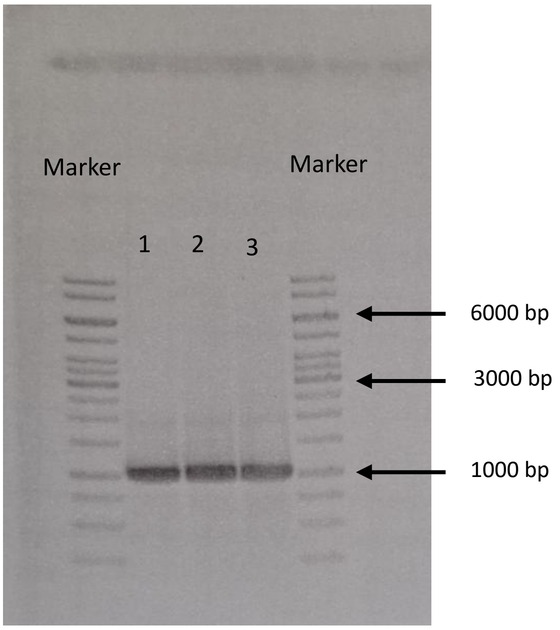

Figure S2: The Agarose gel shows DNA fragments obtained after PCR to verify the presence of pJIR751, related to "Expected Outcomes", Table 2. Marker: GeneRuler 1 kb Thermo Fisher. Lane 1: positive control, 978 bp long fragment, amplified with the primers pJIR751_fwd (gagtcagtgagcgaggaa) and pJIR751_rev (gcaaaaacaggaaggcaa) from the template pJIR751 (expected fragment: 978 bp), isolated from *E. coli* DH5α. Lane 2 and 3: 978 bp long fragment, amplified with the primers pJIR751 pJIR751_fwd (gagtcagtgagcgaggaa) and pJIR751_rev (gcaaaaacaggaaggcaa) from the template pJIR751, isolated from *E. callanderi* (expected fragment: 978 bp).

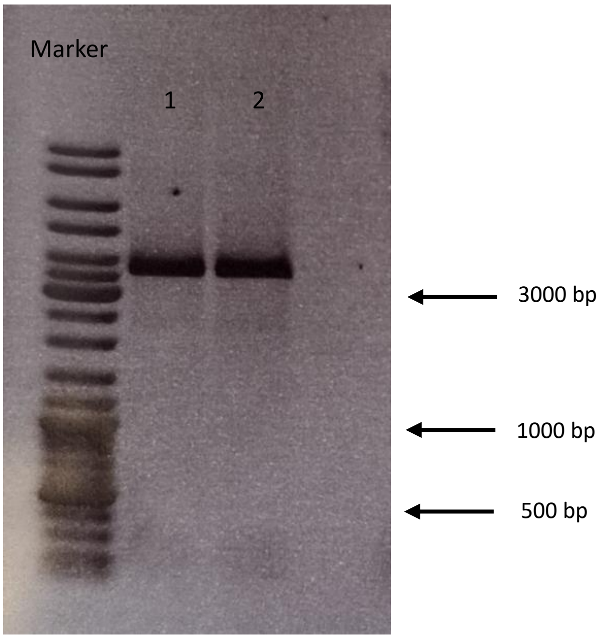

Figure S3: The Agarose gel shows DNA fragments obtained after PCR to verify the presence of MPD23_P_{bgaL}_ldhD, related to "Expected Outcomes", Table 2. Marker: GeneRulerMix Thermo Fisher. Lane 1: positive control, 3395 bp long fragment, amplified with the primers ColE1+tra-F2 ccatcaagaagagcgac and seq_rev ggcttttcatcatcacga from the template MPD23_P_{bgaL}_ldhD, isolated from *E. coli* XL-1 blue (expected fragment: 3395 bp). Lane 2: 3395 bp long fragment, amplified with the primers ColE1+tra-F2 (ccatcaagaagagcgac) and seq_rev (ggcttttcatcatcacga) from the template MPD23_P_{bgaL}_ldhD, isolated from *A. woodii* (expected fragment: 3395 bp).

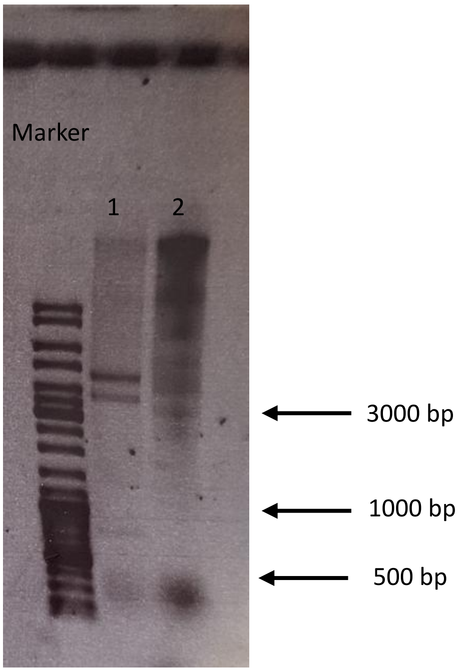

Figure S4: The Agarose gel shows DNA fragments obtained after restriction digestion of the plasmids pMTL83251 and pMTL87151 to verify the presence of pMTL83251 and pMTL87151, related to "Expected Outcomes", Table 2. Marker: GeneRulerMix Thermo Fisher. Lane 1: pMTL83251 and pMTL87151 isolated from *A. woodii* [pMTL83251] [pMTL87151] digested with

the double cutter ApaI. Fragments: 766 bp, 3823 bp, and 4727 bp (expected fragments: 766 bp, 3823 bp, 4727 bp). Lane 2: pMTL83251 and pMTL87151 isolated from *A. woodii* [pMTL83251] [pMTL87151] non-digested.

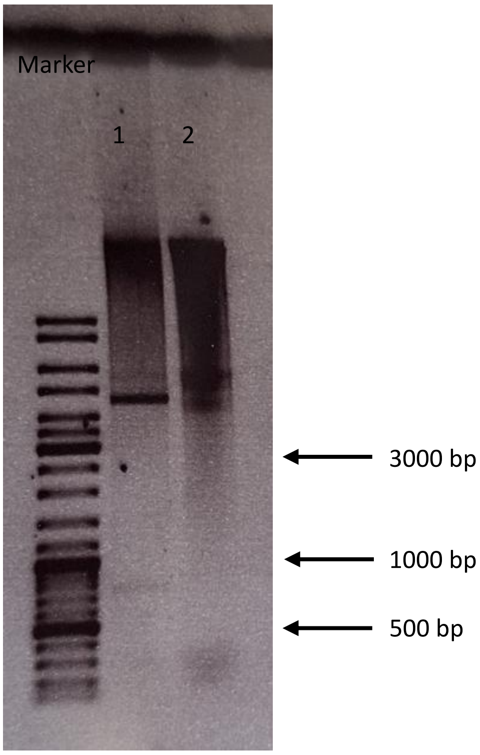

Figure S5: The Agarose gel shows DNA fragments obtained after restriction digestion of the plasmids pMTL87151 to verify the presence of pMTL87151, related to “Expected Outcomes”, Table 2. Marker: GeneRulerMix Thermo Fisher. Lane 1: pMTL87151 isolated from *A. woodii* [pMTL87151] digested with the double cutter ApaI. Fragments: 766 bp and 4727 bp (expected fragments: 766 bp, 4727 bp). Lane 2: pMTL87151 isolated from *A. woodii* [pMTL83251] [pMTL87151] non-digested.

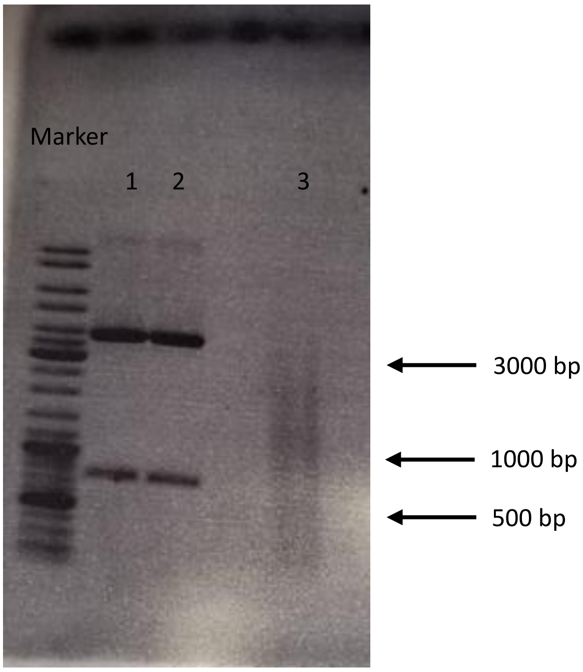

Figure S6: The Agarose gel shows DNA fragments obtained after restriction digestion of the plasmid pMTL83251 to verify the presence of pMTL83251, related to “Expected Outcomes”, Table 2. pMTL83251. Marker: GeneRulerMix Thermo Fisher. Lane 1: pMTL83251 isolated from *E. coli XL-1 blue* [pMTL83251] digested with the double cutter ApaI. Lane 2: pMTL83251 isolated from *A. woodii* [pMTL83251] digested with the double cutter ApaI. Fragments: 766 bp, and 3823 bp (expected fragments: 766 bp, 3823 bp). Lane 3: pMTL83251 isolated from *A. woodii* [pMTL83251] non-digested.

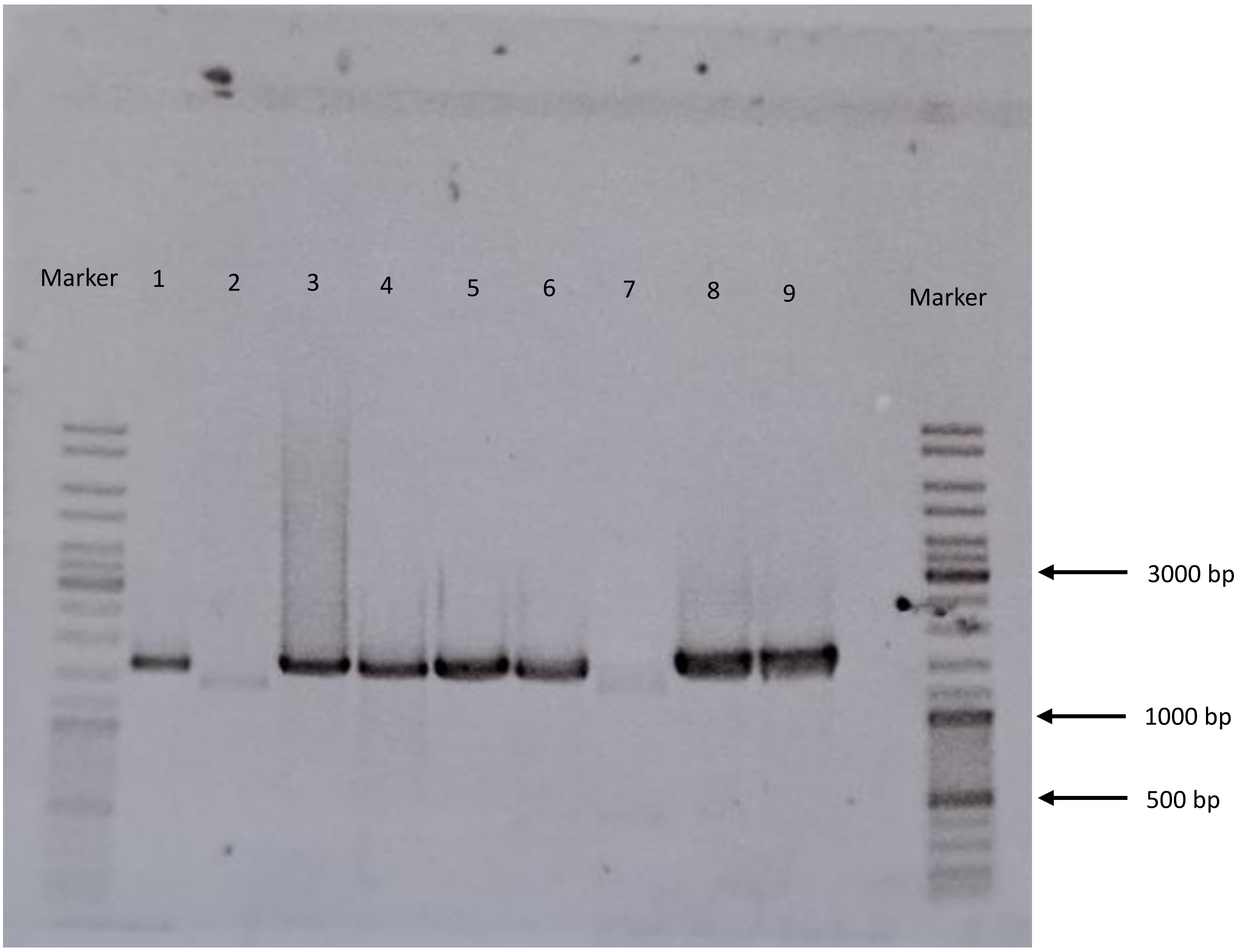

Figure S7: The Agarose gel shows DNA fragments obtained after PCR to verify the presence of pMTL871ksb_Ppta-ack_act_PackA-theo_pfl, related to “Expected Outcomes”, Table 2. Marker: GeneRulerMix Thermo Fisher. Lane 1: Positive control, 1652 bp long fragment, amplified with the primers seq_pfl_Cpas_fwd (gtgatatgctggatgagc) and pfl_rev (gcatgtctgcaggccCTCGAG ttataatttttcatgaaaagttctacttataac) from the template pMTL871ksb_Ppta-ack_act_PackA-theo_pfl (expected fragment: 1652 bp), isolated from *E. coli* XL-1 blue [pMTL871ksb_Ppta-ack_act_PackA-theo_pfl]. Lane 2: Negative control (water). Lane 3 and 4: 1652 bp long fragment, amplified with the primers seq_pfl_Cpas_fwd (gtgatatgctggatgagc) and pfl_rev (gcatgtctgcaggccCTCGAGttataatttttcatgaaaagttctacttataac) from the template pMTL871ksb_Ppta-ack_act_PackA-theo_pfl (expected fragment: 1652 bp), isolated from *A. woodii* [pMTL83251_PbgaL_ldhD] [pMTL871ksb_Ppta-ack_act_PackA-theo_pfl]. Lane 5 and 6: 1652 bp long fragment, amplified with the primers seq_pfl_Cpas_fwd (gtgatatgctggatgagc) and pfl_rev (gcatgtctgcaggccCTCGAGttataatttttcatgaaaagttctacttataac) from the template

pMTL871ksb_Ppta-ack_act_PackA-theo_pfl (expected fragment: 1652 bp), isolated from *A. woodii* [pMTL83251_PlctA_ldhD] [pMTL871ksb_Ppta-ack_act_PackA-theo_pfl]. Lane 7: Negative control (water). Lane 8 and 9: 1652 bp long fragment, amplified with the primers seq_pfl_Cpas_fwd (gtgatatgctggatgagc) and pfl_rev (gcatgtctgcaggccCTCGAG ttataatttttcatgaaaagttctacttataac) from the template pMTL871ksb_Ppta-ack_act_PackA-theo_pfl (expected fragment: 1652 bp), isolated from *A. woodii* [pMTL871ksb_Ppta-ack_act_PackA-theo_pfl].

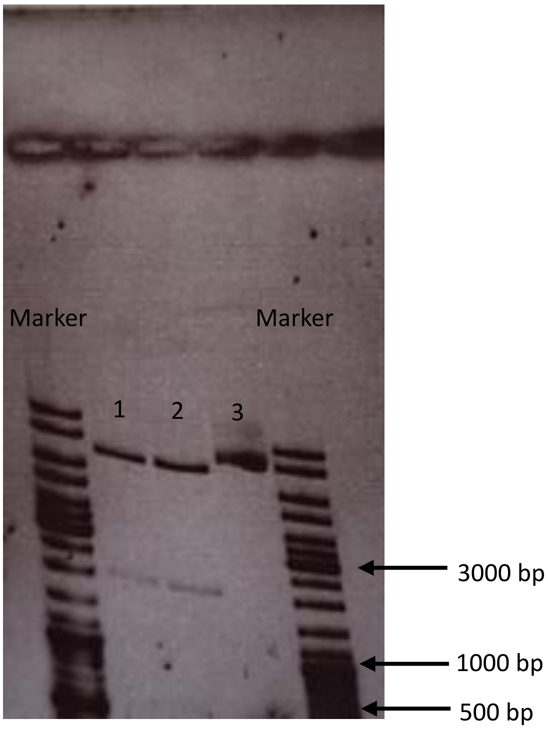

Figure S8: The Agarose gel shows DNA fragments obtained after restriction digestion of the plasmid MPD21 to verify the presence of MPD21, related to “Expected Outcomes”, Table 2. MPD21. Marker: GeneRulerMix Thermo Fisher. Lane 1: MPD21 isolated from *E. coli* XL-1 blue [MPD21] digested with the enzymes SacI and SmaI. Fragments: 2057 bp and 7258 bp (expected fragments: 2057 bp, 7258 bp). Lane 2: MPD21 isolated from *A. woodii* [MPD21] digested with the enzymes SacI and SmaI. Fragments: 2057 bp and 7258 bp (expected fragments: 2057 bp, 7258 bp). Lane 3: MPD21 isolated from *A. woodii* [MPD21] non-digested.
